# The Gene Rearrangement, Loss, Transfer, and Deep Intronic Variation in Mitochondrial Genomes of *Conidiobolus*

**DOI:** 10.3389/fmicb.2021.765733

**Published:** 2021-11-11

**Authors:** Yong Nie, Heng Zhao, Zimin Wang, Zhengyu Zhou, Xiaoyong Liu, Bo Huang

**Affiliations:** ^1^Anhui Provincial Key Laboratory for Microbial Pest Control, Anhui Agricultural University, Hefei, China; ^2^School of Civil Engineering and Architecture, Anhui University of Technology, Ma’anshan, China; ^3^School of Ecology and Nature Conservation, Institute of Microbiology, Beijing Forestry University, Beijing, China; ^4^College of Life Sciences, Shandong Normal University, Jinan, China; ^5^State Key Laboratory of Mycology, Institute of Microbiology, Chinese Academy of Sciences, Beijing, China

**Keywords:** basal fungi, Entomophthorales, mitogenome, intron, gene order

## Abstract

The genus *Conidiobolus* s.s. was newly delimited from *Conidiobolus* s.l. In order to gain insight into its mitochondrial genetic background, this study sequenced six mitochondrial genomes of the genus *Conidiobolus* s.s. These mitogenomes were all composed of circular DNA molecules, ranging from 29,253 to 48,417 bp in size and from 26.61 to 27.90% in GC content. The order and direction for 14 core protein-coding genes (PCGs) were identical, except for the *atp8* gene lost in *Conidiobolus chlamydosporus*, *Conidiobolus polyspermus*, and *Conidiobolus polytocus*, and rearranged in the other *Conidiobolus* s.s. species. Besides, the *atp8* gene split the *cox1* gene in *Conidiobolus taihushanensis*. Phylogenomic analysis based on the 14 core PCGs confirmed that all *Conidiobolus* s.s. species formed a monophyly in the *Entomophthoromycotina* lineage. The number and length of introns were the main factors contributing to mitogenomic size, and deep variations and potential transfer were detected in introns. In addition, gene transfer occurred between the mitochondrial and nuclear genomes. This study promoted the understanding of the evolution and phylogeny of the *Conidiobolus* s.s. genus.

## Introduction

As the largest group in the family Ancylistanceae (Entomophthorales), the *Conidiobolus* s.l. has a long list of 80 records in the Index Fungorum^1^. Most *Conidiobolus* s.l. species are saprobic and widely distributed in plant debris and soil, while some members are parasitic to insects (Humber, 2012; Gryganskyi et al., 2013; Nie et al., 2020b), and a few species were proposed as pathogens of mammals and humans (Vilela et al., 2010; Vilela and Mendoza, 2018). A recent multi-locus phylogenetic analysis divided the genus *Conidiobolus* s.l. into four lineages (Nie et al., 2020b), and consequently, four genera *Capillidium*, *Conidiobolus* s.s., *Microconidiobolus*, and *Neoconidiobolus* were delimited. The genus *Conidiobolus* s.s. holds 17 species only and is characterized by microspores arising from conidia (Nie et al., 2020a,b). Currently, four nuclear and four mitochondrial genomes of *Conidiobolus* s.l. are available in GenBank^2^ and JGI,^3^ involving only one mitogenome of the *Conidiobolus* s.s. Remarkably, *Capillidium heterosporum* was the first fungus whose nuclear genome harbors the gene families CYP5014 and CYP5136 encoding cytochrome P450s, and it contained the second greatest number of introns among basal fungi (Nie et al., 2019; Wang et al., 2020).

Mitochondria of eukaryotes were considered to be evolved from endosymbiotic Alphaproteobacteria (Lang et al., 1999). As an important organelle in the eukaryotic cell, mitochondria are in charge of energy production through oxidative phosphorylation and cellular respiration (Henze and Martin, 2003; Chan, 2006; Martin et al., 2015). Except for the anaerobic mitochondrion-free *Neocallimastigomycota*, fungal mitogenomes have a wide range of sizes (Aguileta et al., 2014; Sandor et al., 2018). As reported, the smallest is 12,100 bp in the basal fungus *Rozella allomycis* (James et al., 2013), and the largest is 287,403 bp of an ectomycorrhizal fungus *Tuber calosporum* (Li X. et al., 2020). Most fungi possess a circular mitochondrial genome (Sandor et al., 2018), and a few are known as linear such as *Kluyveromyces marxianus* (Inokuma et al., 2015) and *Synchytrium endobioticum* (van de Vossenberg et al., 2018). Fungal mitogenomes typically harbor indefinite numbers of transfer RNA genes (*trn*), two ribosomal RNA genes (*rnl* and *rns*), and 14 core protein-coding genes (PCGs) for NADH dehydrogenase subunits (*nad1*, *nad2*, *nad3*, *nad4*, *nad4L*, *nad5*, and *nad6*), cytochrome oxidase subunits (*cox1*, *cox2*, and *cox3*), apocytochrome b (*cob*), and ATP synthase subunits (*atp6*, *atp8*, and *atp9*) (Lang, 2018). A few of these genes are sometimes lost (James et al., 2013), while two other genes encoding the ribosomal protein S3 (*rps3*) and the RNA subunit of RNase P (*rnpB*) are present in certain fungal mitogenomes (Franco et al., 2017).

Aside from genomic size and gene organization, fungal mitogenome comparative analyses have widely paid attention to intron distribution and variation (Nadimi et al., 2016; Nie et al., 2019; Zhang et al., 2019; Li Q. et al., 2020). The number and length of introns vary and contribute to mitogenome size (Friedrich et al., 2012; Torriani et al., 2014). For instance, *Clavaria fumosa* mitogenome contains 93,365 bp of intronic sequence, accounting for 36.36% (Wang et al., 2020). Two groups (I and II) of mitochondrial introns were recognized based on secondary structure and splicing mechanism (Saldanha et al., 1993). Group I mitochondrial introns frequently exist in fungal mitogenomes, while group II introns dominate in plant mitogenomes (Lang et al., 2007). Group I introns are phylogenetically divided into six main subgroups (IA, IA3, IB, IC1, IC2, and ID), with open reading frames (ORFs) usually coding homing endonucleases (HEs: LAGLIDADG and GIY-YIG). Mitogenomic introns also vary in position among species and even isolates and, consequently, are divided into different position classes (Pcls) (Beaudet et al., 2013; Wang et al., 2018). Recently, a novel nomenclature of mitochondrial introns has been proposed to avoid confusion when comparing different fungal mitogenomes, and the nomenclature system was based on: (1) three-letter abbreviation of host scientific name; (2) host gene name; (3) one capital letter P (for group I introns), S (for group II introns), or U (for introns unclassified); and (4) intron insertion site in the host gene according to the cyclosporin-producing fungus *Tolypocladium inflatum* (Zhang and Zhang, 2019). In general, mitogenomic introns are highly similar and homologous in the same Pcl (Ferandon et al., 2010) and have an implication for inferring fungal evolution (Sandor et al., 2018). Besides the above characteristics, the codon usage, repetitive elements, and tRNA types have also been used to evaluate the evolutionary dynamics of the fungal mitogenomes (Yuan et al., 2017; Abboud et al., 2018; Li Q. et al., 2020, Li et al., 2021). Mitochondrial genomes are inherited by a single parent and have a higher number of copies, causing a faster evolution than nuclear (Ballard and Whitlock, 2004; Pantou et al., 2006; Cameron, 2014). Therefore, the core conserved PCGs were always used for phylogenetic analyses in both basal and higher fungi (Zhang et al., 2017; Nie et al., 2019; Cai et al., 2021; Li et al., 2021).

According to the NCBI database,^4^ only 29 complete mitogenomes of basal fungi were available. In this study, we reported and performed comparative analyses of six additional basal fungi including *Conidiobolus chlamydosporus*, *Conidiobolus lichenicolus*, *Conidiobolus mycophagus*, *Conidiobolus polyspermus*, *Conidiobolus polytocus*, and *Conidiobolus taihushanensis*. The aims of this study are: (1) to characterize the organization, codon usage, and repetitive element of *Conidiobolus* s.s. mitogenomes, (2) to reveal the diversity of intron and intronic ORFs among *Conidiobolus* s.s. mitogenomes, and (3) to investigate the taxonomic status of *Conidiobolus* s.s. in the subphylum *Entomophthoromycotina* based on the concatenated amino-acid sequences of 14 PCGs in mitogenomes. This work will promote the understanding of origin, evolution, and phylogeny of entomophthoroid fungi and related species.

## Materials and Methods

### Fungal Isolates and DNA Extraction

Six ex-types of *Conidiobolus* s.s. were obtained from the American Type Culture Collection, Manassas, United States (ATCC) and the China General Microbiological Culture Collection Center, Beijing, China (CGMCC) and duplicated in the Research Center for Entomogenous Fungi, Anhui Agricultural University, Hefei, Anhui, China (RCEF): ATCC 12242 = RCEF 6832 (*C. chlamydosporus*), ATCC 12244 = RCEF 6833 (*C. polytocus*), ATCC 14444 = RCEF 6834 (*C. polyspermus*), ATCC 16200 = RCEF 6835 (*C. lichenicolus*), ATCC 16201 = RCEF 6836 (*C. mycophagus*), and CGMCC 3.16016 = RCEF 6559 (*C. taihushanensis*). Fresh fungal biomass on potato dextrose agar (PDA: potato 200 g, dextrose 20 g, agar 20 g, H_2_O 1,000 ml) at 21°C for 7 days was collected. Genomic DNA was extracted using the CTAB method with liquid nitrogen grinding (Watanabe et al., 2010).

### Mitogenome Sequencing, Assembly, and Annotation

Whole mitogenome sequencing of six *Conidiobolus* s.s. species was performed using an Illumina HiSeq X Ten sequencing platform (Nextomics Biosciences, Co., Ltd., Wuhan, China). The paired-end libraries with 300 bp inserts were constructed according to the manufacturer’s instruction (AIR Paired-End DNA Sequencing Kit; Bioo Scientific). Raw sequencing data were evaluated by FastQC 0.11.8 (Andrews, 2010) for quality, GC content, and length. Trimmomatic 0.39 (Bolger et al., 2014) was used to remove the adapter reads and filter low-quality sequences, resulting in a size of 15–18 Gb data per ex-type strain. The mitogenomes were assembled by Norgal 1.0 (Alnakeeb et al., 2017) from clean data without reference sequences and rechecked by NOVOPlasty 4.2 (Dierckxsens et al., 2017) with *C. heterosporum* (NC_040967) as reference sequence. The assembled mitogenomes were annotated automatically with the MFannot tool^5^ using the mitochondrial genetic code (also known as genetic code 4) and GeSeq (Tillich et al., 2017) to predict mitogenome organization (Zhang et al., 2017; Nie et al., 2019; Li Q. et al., 2020). Then, the boundaries of rRNAs were rechecked by aligning to other reported mitogenomes of entomophthoroid fungi including *C. heterosporum* (NC040967), *Neoconidiobolus thromboides* (MW795364), and *Microconidiobolus nodosus* (MW795365) (Nie et al., 2019, 2021; Cai et al., 2021). The transfer-RNA (tRNA) annotations were confirmed by tRNAscan-SE 1.21 (Schattner et al., 2005). Intronic and intergenic spacers were searched by ORF Finder^6^ and nucleotide 6-frame translation-protein BLAST (blastx, see text footnote 6). Exonerate v2.2 (Slater and Birney, 2005) was used to verify intron–exon borders of PCGs. Circular mitogenome maps were drawn with OGDraw v1.3.1 (Lohse et al., 2013; Greiner et al., 2019) after manual proofreading.

### Comparative Analysis of Mitogenomes in *Conidiobolus* s.s.

The base compositions of the six *Conidiobolus* s.s. mitogenomes were tested by DNASTAR Lasergene v7.1.^7^ Strand asymmetries were calculated according to the following formulas: AT skew = [A - T]/[A + T] and GC skew = [G - C]/[G + C] (Li et al., 2021). The most frequently used codons and amino acids were predicted using the Sequence Manipulation Suite (Stothard, 2000). The pairwise genetic distances between each pair of the 13 core PCGs (excluding *atp8*) in the six *Conidiobolus* s.s. mitogenomes were detected using MEGAX based on the Kimura-2-parameter (K2P) substitution model (Kumar et al., 2018). The non-synonymous substitution rates (Ka) and synonymous substitution rates (Ks) were calculated under DnaSP v6.10.01 (Rozas et al., 2017). BLASTN searches were conducted to investigate the lateral gene transfer (LGT) between mitochondrial and nuclear genomes (Unpublished). Tandem repeats were analyzed using Tandem Repeat Finder with default parameters (Benson, 1999). The online analysis of SSRIT^8^ was used to identify simple sequence repeats (SSRs) by allowing the maximum motif-length group of decamer and the minimum repeat number of five. A gene synteny analysis was performed with Mauve (Darling et al., 2004) under default alignment parameters for the six *Conidiobolus* s.s. mitogenomes obtained herein and four mitogenomes of related species reported elsewhere (*C. heterosporum*, *Conidiobolus* sp., *N. thromboides*, and *M. nodosus*).

### Intron Analysis

A novel nomenclature of mitochondrial introns was followed, and only intronic ORFs >300 bp were considered (Zhang et al., 2017, 2019). To reveal the intron evolution in the family Ancylistanceae, introns in core PCGs of six *Conidiobolus* s.s. and four related species mitogenomes were classified into different Pcls according to previously described methods (Ferandon et al., 2013). The Pcls of core PCGs in Ancylistanceae were named according to the insert position in the reference mitogenome of *C. heterosporum* (NC_040967) (Nie et al., 2019). Pcls had the same insertion site considered homologous, usually highly similar in sequence (Ferandon et al., 2010).

### Phylogenomic Analysis

To detect the phylogenetic relationship of the six *Conidiobolus* s.s. in the basal fungi, all 14 conserved PCGs were used. A total of 38 fungal mitogenomes were downloaded from GenBank for comparison, and three plant and animal mitogenomes served as outgroups (Supplementary Table 1). The amino acid sequences of each locus were aligned with MAFFT v6.864 (Katoh and Standley, 2013) and concatenated with SequenceMatrix 1.7.8 (Vaidya et al., 2011). Phylogenetic trees were constructed by Maximum Likelihood (ML) and Bayesian Inference (BI) using the best model of amino acid assessed with Akaike Information Criterion (AIC) in Modeltest 3.7 (Posada and Crandall, 1998). Under the GTR + I + G model, the ML phylogram was constructed using RAxML 8.1.17 with 1,000 bootstrap replicates (Stamatakis, 2014). The BI analysis was processed with 500,000 generations and four chains (one cold and three hot) using MrBayes 3.2.2 (Ronquist and Huelsenbeck, 2003). Markov Chain Monte Carlo (MCMC) chains ran until the convergences met and the standard deviation fell below 0.01. The first 25% of the generations were discarded as “burn-in,” and a posterior probability was estimated for the remaining sampled generations.

### Data Availability

The six mitogenome sequences of *Conidiobolus* s.s. were deposited in GenBank, and their accession numbers are listed in Table 1.

**TABLE 1:** Mitogenome information of six *Conidiobolus* s.s. species.

		***C. chlamydosporus***	***C. lichenicolus***	***C. mycophagus***	***C. polyspermus***	***C. polytocus***	***C. taihushanensis***
Accession number	Mitogenomes	MZ436168	MZ436169	MZ436170	MZ436171	MZ436172	MZ436173
GC content	Mitogenomes	26.61%	27.31%	27.87%	27.90%	26.72%	27.03%
Lengths (bp)	Mitogenomes	29,253	42,713	32,218	32,900	43,599	48,417
	rRNA genes	3,903	3,936	3,917	3,975	3,930	3,923
	tRNA genes	1,791	1,719	1,719	1,719	1,718	1,793
	Intergenic ORFs	1,365	6,819	4,986	4,683	2,808	7,959
	PCG exons	10,730	12,516	13,488	12,477	12,186	12,306
	PCG introns	6,905	14,470	6,837	7,186	21,084	19,835
	Intronic ORFs	3,975	11,388	5,118	5,097	13,899	13,824
Numbers	rRNA genes	2	2	2	2	2	2
	tRNA genes	24	23	23	23	23	24
	Intergenic ORFs	1	9	6	5	3	8
	PCG exons	21	28	21	20	38	39
	PCG introns	9	14	6	7	25	25
	Intronic ORFs	5	13	6	6	16	18
Percentages	rRNA genes	13.34%	9.21%	12.16%	12.08%	9.01%	8.10%
	tRNA genes	6.12%	4.02%	5.34%	5.22%	3.94%	3.70%
	Intergenic ORFs	4.67%	15.96%	15.48%	14.23%	6.44%	16.44%
	PCG exons	36.68%	29.30%	41.86%	37.92%	27.95%	25.42%
	PCG introns	23.60%	33.88%	21.22%	21.84%	48.36%	40.97%
	Intronic ORFs	13.59%	26.66%	15.89%	15.49%	31.88%	28.55%

## Results

### Characterization of the Six *Conidiobolus* s.s. Mitogenomes

**FIGURE 1:**
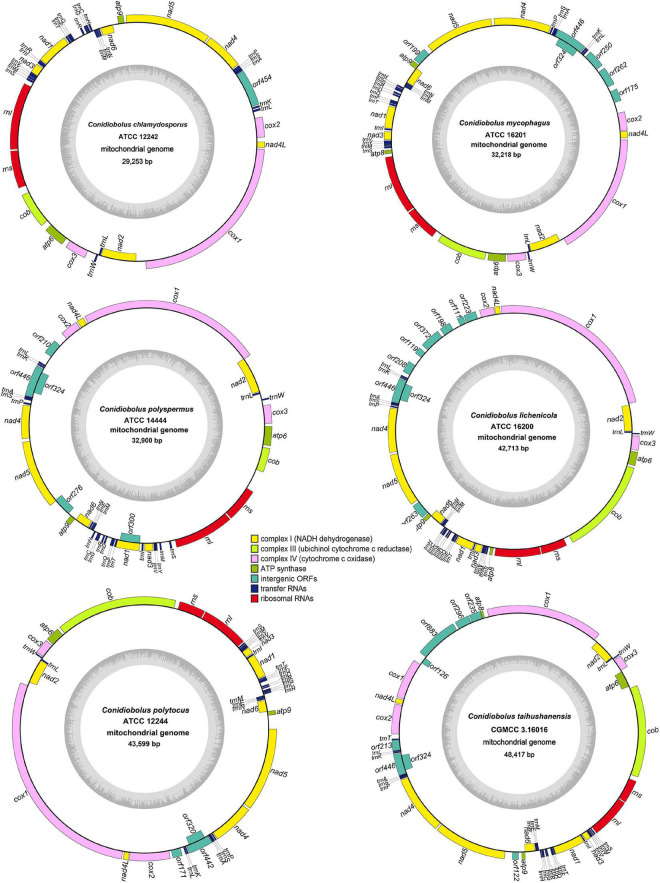
Circular complete mitogenome maps of six ex-types in *Conidiobolus* s.s. genes are represented by different colored blocks. Genes outside and inside the outermost black ring line are transcribed in a clockwise and counter-clockwise direction, respectively. Inner gray rings show GC contents.

**FIGURE 2:**
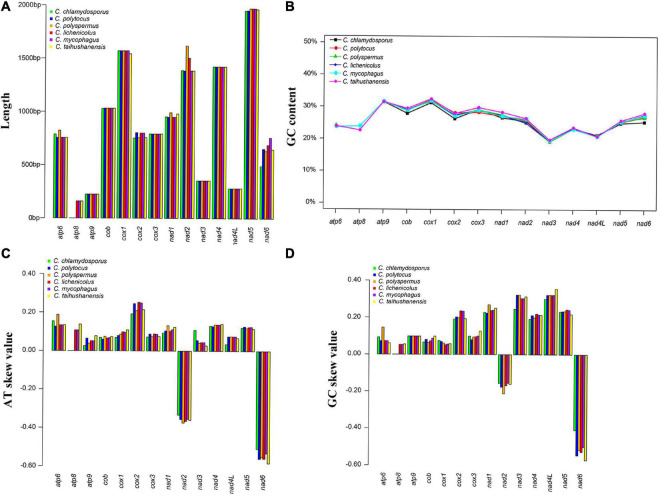
Variations of 14 protein-coding genes (PCGs) in the six *Conidiobolus* s.s. mitogenomes. **(A)** Length; **(B)** GC content; **(C)** AT skews; **(D)** GC skews.

**FIGURE 3:**
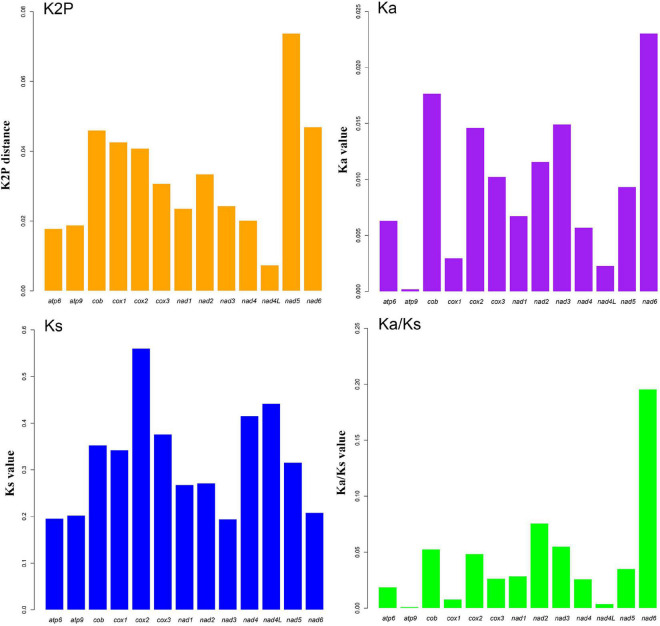
Genetic analysis of 13 protein-coding genes (PCGs) in six *Conidiobolus* s.s. mitogenomes. K2P, the Kimura-2-parameter distance; Ka, the mean number of non-synonymous substitutions per non-synonymous site; Ks, the mean number of synonymous substitutions per synonymous site. Gene *atp8* is not included due to its loss in three species.

**FIGURE 4:**
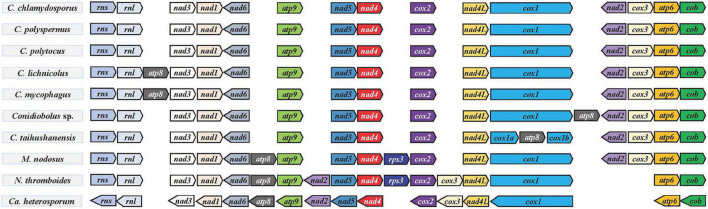
Gene order in *Conidiobolus* s.s. mitogenomes and four related species. All species were adjusted to start with the gene *rns*. Genes on the forward and reverse strands were indicated by right and left arrows, respectively.

The complete circular mitogenomes of the six *Conidiobolus* s.s. were assembled (Figure 1 and Table 1). With a wide length range of 29,253–48,417 bp and a narrow GC content range of 26.61–27.90%, a single mitogenome contained 1–9 intergenic ORFs and a conserved set of 38–40 genes including two rRNA genes, 23–24 tRNA genes, and 13–14 PCGs. A total of 26 types of intergenic ORFs were found in all the six *Conidiobolus* s.s. mitogenomes: 24 were detected once, and two (*orf324* and *orf446*) were examined four times (Supplementary Table 2). A total of 23 types of tRNA genes encoded 20 standard amino acids. The tRNA gene *trnM(cat)* had two copies in every mitogenome, while the tRNA gene *trnT(tgt)* had two copies in the mitogenome of *C. taihushanensis*. Two types of anticodons for each of the genes *trnL* and *trnS* were presented in mitogenome of every species, while the *trnR* gene with two types of anticodons was presented in *C. chlamydosporus* (Supplementary Table 3). Almost all 14 conserved PCGs existed in all species, except the *atp8* gene not found in *C. chlamydosporus*, *C. polyspermus*, and *C. polytocus* (Figures 1, 2, 4). Exons of these PCGs had a length range of 10,730–13,488 bp and a GC content range of 25.42–41.86%. AT and GC skews were both positive in most conserved PCGs but negative in genes *nad2* and *nad6* (Figure 2). The genes *nad5* and *nad4L* had the largest and smallest K2P genetic distances, respectively (Figure 3). The Ka values of *nad6* and *atp9* were the largest and smallest in the six *Conidiobolus* s.s. species, respectively. The *cox2* gene had the largest synonymous substitutions rate (Ks), while *nad3* had the smallest. Ka/Ks values were all <1.00. The highest Ka/Ks value was observed for *nad6*, while *atp9* exhibited the lowest Ka/Ks value.

### Gene Order in *Conidiobolus* s.l.

The order of PCGs and rRNA genes was compared among *Conidiobolus* s.l. mitogenomes including seven *Conidiobolus* species and three allied species *C. heterosporum* (NC040967), *M. nodosus* (MW795365), and *N. thromboides* (MW795364) (Figure 4). The result showed that most genes were arranged in the same order on the same strand, whereas genes *nad2* and *nad6* were on the other strand. Furthermore, all genes of *C. heterosporum* were on the other strand. Other changes involved the gene loss of *rps3* in almost all *Conidiobolus* s.l. except *M. nodosus* and *N. thromboides*, the gene *atp8* lost in three species (*C. chlamydosporus*, *C. polyspermus*, and *C. polytocus*) and rearranged in the other four *Conidiobolus* species, the gene rearrangement of *nad2* and *cox3* in *N. thromboides* and *C. heterosporum*, and the gene split of *cox1* by *atp8* in *C. taihushanensis*.

**FIGURE 5:**
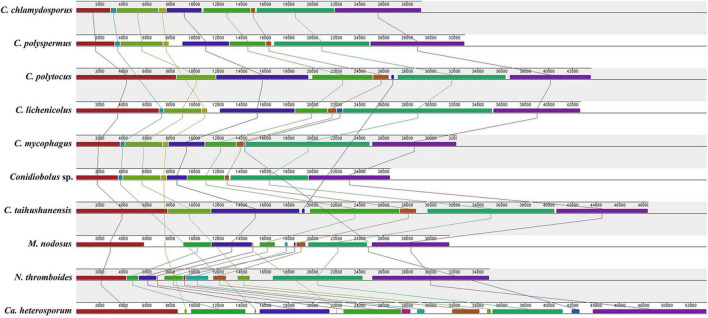
Synteny comparison of mitogenomes among six *Conidiobolus* s.s. and four related species.

A similar synteny was revealed for the group of *Conidiobolus* spp. and *M. nodosus*, and another similar synteny was revealed for the other group of *N. thromboides* and *C. heterosporum*. Between these two groups was an obvious rearrangement (Figure 5).

### Introns and Intronic Open Reading Frames in *Conidiobolus* s.l.

Seven of the 14 conserved PCGs of the six *Conidiobolus* s.s. mitogenomes assembled herein had no introns and intronic ORFs. The other seven PCGs contained introns and intronic ORFs (Supplementary Table 4). They totally possessed 86 introns, but varied remarkably among species in number, ranging from 6 in *C. mycophagus* to 25 in *C. polytocus* and *C. taihushanensis*, as well as in length percentage, ranging from 17.00% in *C. mycophagus* to 42.60% in *C. polytocus* (Tables 1, 2). These introns were annotated to three subgroups IA, IB, and ID of the group I type, except three unknown each in the *cob*, *nad1*, and *nad5* genes of *C. polytocus* and one unknown in the *cox1* gene of *C. taihushanensis* (Supplementary Table 4).

**TABLE 2:** Introns in *Conidiobolus* s.s. mitogenomes.

**Taxa**	**No. of introns**	**Protein-coding genes (no. of introns)**													
		***atp6***	***atp8***	***atp9***	***cob***	***cox1***	***cox2***	***cox3***	***nad1***	***nad2***	***nad3***	***nad4***	***nad4L***	***nad5***	***nad6***
*C. chlamydosporus*	9	0	–	0	1	5	0	0	1	0	0	0	0	2	0
*C. lichenicolus*	14	0	0	0	4	8	0	0	0	0	0	1	0	1	0
*C. mycophagus*	6	0	0	0	1	3	0	0	0	0	0	1	0	1	0
*C. polyspermus*	7	0	–	0	0	6	0	0	0	0	0	0	0	1	0
*C. polytocus*	25	0	–	0	7	9	2	0	2	0	0	1	0	4	0
*Conidiobolus* sp.	6	0	0	0	1	3	0	0	0	0	0	0	0	2	0
*C. taihushanensis*	25	1	0	0	6	10	1	0	2	0	0	2	0	3	0
Total	86	1	0	0	19	41	3	0	5	0	0	5	0	12	0

*“–” means that the gene is not annotated.*

In total, 64 intronic ORFs were detected in the six *Conidiobolus* mitogenomes (Table 1 and Supplementary Table 4). These intronic ORFs were mainly encoded for LAGLIDADG and GIY-YIG endonucleases, and most were specific. Only two intronic ORFs (*orf327* and *orf290*) were shared by three species, and seven (*orf217*, *orf291*, *orf295*, *orf298*, *orf328*, *orf331*, and *orf333*) were shared by two species.

Altogether, 77 Pcls were detected in nine conserved PCGs in all the 10 *Conidiobolus* s.l. species, with 31 in the *cox1* gene and 19 in the *cob* gene. Only one Pcl was detected in each of the *atp9* and *cox3* genes (Supplementary Figure 1). Pcls P9, P12, P19, and P30 were widely distributed in the *cox1* gene in more than five species. Pcl P6 was the most widely distributed in the *cob* gene.

### Codon Usage and Repeat Elements in *Conidiobolus* s.l.

Most conserved PCGs in the *Conidiobolus* s.s. mitogenomes started with ATG (Supplementary Table 5), except *atp6* in *C. polyspermus* (GTG), *cox2* in *C. lichenicolus*, *C. mycophagus*, and *C. polytocus* (GTG), *nad2* in *C. polyspermus* (TTG), and *nad6* in *C. mycophagus* (TTG). The TAA was the most commonly used stop codon, and AAA, TAG, and TAA were also used. The most frequently used codons were ATA (isoleucine; Ile), AAT (asparagine; Asn), TTA (leucine; Leu), TCA (serine; Ser), and GGT (glycine; Gly) (Supplementary Figure 2).

Variation of repeat sequence regions among *Conidiobolus* s.l. mitogenomes ranged from 1 to 30 in number and 45 to 1,904 bp in length (Supplementary Table 6). A total of 111 types of tandem repeat motifs varied from 17 to 55 in number, 35 to 293 bp in length, and 80.42 to 100% in nucleotide similarity (Supplementary Table 7). The longest tandem repeat with 200 bp was found in *C. chlamydosporus*, which was located in the protein-coding regions of *orf454*. In all, there were 83.78% tandem repeat sequences that were repeated two to six times in the mitogenome of six *Conidiobolus* s.s., and the highest copy number was 24.7 in the *C. chlamydosporus* mitogenome. A total of 50 SSRs were identified in six *Conidiobolus* s.s. mitogenomes. The dimer (AT or TA) and trimer (GAA, TAT, ATT, and AAT) motifs were the most commonly detected types, while four (AATA, seven repeats) and seven (AAAAAAC, five repeats) motifs were only detected in *C. chlamydosporus* mitogenome (Supplementary Table 8).

Gene transfer analyses showed that 62–150 fragments were identified with a total percent identity from 81.29 to 100% in the six *Conidiobolus* s.s. mitogenomes (Supplementary Table 9). Correspondingly, the total alignment sequences ranged from 21,708 to 39,339 bp. The most fragment was detected in the *C. taihushanensis* mitogenome. The largest aligned fragment with 2,536 bp was located between *cox1* and *nad4L* genes in the *C. chlamydosporus* mitogenome. The similarity of this large alignment was 99.65% compared with its nuclear genome, with five mismatches and four gaps.

### Phylogenomic Analyses for *Conidiobolus* s.l.

**FIGURE 6:**
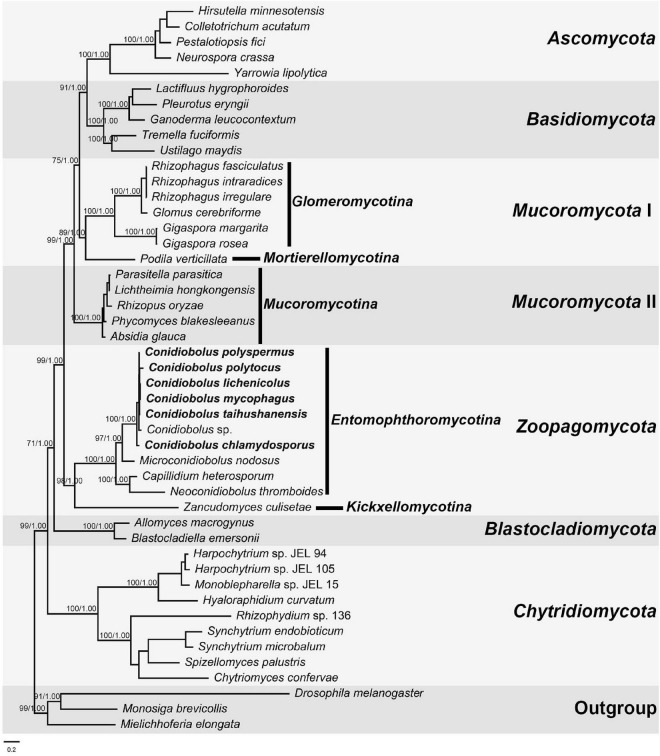
The Maximum Likelihood (ML) phylogenomic tree of fungi based on 14 protein-coding genes (PCGs): *cob*, *cox1*, *cox2*, *cox3*, *nad1*, *nad2*, *nad3*, *nad4*, *nad4L*, *nad5*, *nad6*, *atp6*, *atp8*, and *atp9*. Support values for ML and Bayesian analysis (BI) greater than 70% and 0.95, respectively, are given on relative clade. *Drosophila melanogaster*, *Mielichhoferia elongata*, and *Monosiga brevicollis* serve as outgroups. The lower left bar represents 0.2 substitutes per site.

The combined dataset was composed of 5,941 characters and 47 taxa. The best model applied in the ML and BI analyses was GTR + I + G. The final average standard deviation of split frequencies was 0.000748. The ML and BI phylogenetic trees shared a similar topology, and the ML tree was presented with ML bootstrap/BI posterior probability values of robust clades at relative branches (Figure 6). The seven *Conidiobolus* s.s. species formed a monophyletic group (100/1.00). Three related *Conidiobolus* s.l. species *C. heterosporum*, *M. nodosus*, and *N. thromboides* were basal to the seven *Conidiobolus* s.s. species within the *Entomophthoromycotina* lineage.

## Discussion

Fungal mitogenomes differ greatly in gene content, gene arrangement, repeat elements, and intronic content among species and even closely related ones (Sandor et al., 2018; Li Q. et al., 2020, Li et al., 2021). These differences contain important information about the origin and evolution of species (Sankoff et al., 1992). In this article, we focused on these characteristics to evaluate the evolution of *Conidiobolus* s.s.

The seven *Conidiobolus* s.s. mitogenomes (26,612–48,417 bp) were moderate in size compared with those of other basal fungi (12,100–225,604 bp; Nie et al., 2019). The size of mitogenomes was mainly determined by its introns and intergenic regions (Table 1) as denoted elsewhere (Sandor et al., 2018; Liu W. et al., 2020). A much wider range was observed in a single genus of higher fungi. Taking *Schizosaccharomyces* as an example, its mitogenome sizes ranged from approximately 19,000–80,000 bp (Bullerwell et al., 2003). The wide size range of fungal mitogenomes might imply a variable function in oxidative phosphorylation and cellular respiration.

All the 14 conserved PCGs were all detected in the *Conidiobolus* s.s. mitogenomes, except that the *atp8* gene was absent in the mitogenomes of *C. chlamydosporus*, *C. polyspermus*, and *C. polytocus*. This kind of gene loss is not uncommon. The *atp9* gene was lost from the *Trichoderma gamsii* mitogenome (Kwak, 2021). The *nad3* gene was not annotated in the *Colletotrichum lindemuthianum* mitogenome (De-Queiroz et al., 2018), neither was other NADH genes in some yeast mitogenomes (Friedrich et al., 2012). Most scholars considered that some mitochondrial genes have been transferred to the nuclear genome during the evolution of eukaryotes (Adams and Palmer, 2003). The arrangement of mitochondrial genes in fungal mitogenomes reflected their phylogenetic relationship to certain degrees (Zhang et al., 2018; Wang et al., 2020). As expected, the gene orders were identical in the monophyletic *Conidiobolus* s.s., which was confirmed by the phylogenomic analyses (Figure 6). The gene direction in *C. heterosporum* mitogenome was almost opposite to others, implying its distant phylogenetic relationship to other species. As a complement to gene order analysis, the mitogenomic synteny analysis showed more detail about gene length and rearrangement (Figure 6). Basal fungal groups frequently exhibited intra-genus gene rearrangements (Li et al., 2019, 2021; Zhang et al., 2020), similar to the result herein. It is worth to note that the *cox1* gene in *C. taihushanensis* mitogenome was split by the *atp8* gene. This phenomenon often appeared in basal fungi, especially in the mitogenomes of Glomeromycetes (Pelin et al., 2012; Nadimi et al., 2016; Arcila-Galvis et al., 2021). It may be a long size and abundant introns of *cox1* gene that caused a trans-splicing of *cox1* genes located on different RNA molecules and transcribed separately (James et al., 2013; Nie et al., 2019). It is also worth to note that the *rps3* gene was exhibited in *M. nodosus* and *N. thromboides* mitogenomes. The *rps3* gene encodes a key protein component of the ribosome for protein translation, and it is often fused with an *rnl* intron that harbors an ORF homologous to HEs or a partial sequence of *cox1* gene (Bullerwell et al., 2000; Sethuraman et al., 2009; Franco et al., 2017; Zhang et al., 2019).

As shown in Figure 2, the lengths of *atp6*, *cox2*, *nad1*, *nad2*, and *nad6* varied among the six *Conidiobolus* s.s. mitogenomes species. The GC contents of all PCGs varied across the six mitogenomes, which indicated that the conserved PCGs of *Conidiobolus* s.s. mitogenomes mutate frequently. The *nad4L* gene bore the smallest pairwise genetic distance, indicating that it was highly conserved. The Ka/Ks values of the 13 PCGs were all <1, indicating that these genes were subjected to purifying selection (Figure 3).

A total of 41 introns were detected in the *cox1* gene that occupied mostly half of the total introns in the *Conidiobolus* s.s. mitogenomes (Tables 1, 2). Usually, the *cox1* gene contains many introns in fungal mitogenomes causing its large variation, and it was not suitable as DNA barcode to identify fungal species (Freel et al., 2015; Nadimi et al., 2016; Sandor et al., 2018). Pcls analysis of *cox1* gene and other core PCGs was often used to evaluate the dynamics of introns in basidiomycetes mitogenomes. For example, two large-scale intron loss events were detected in the evolution of Boletales species based on the Pcls analysis of the *cox1* gene (Li et al., 2021). The Pcls analysis of several core PCGs that contained introns was conducted in 27 *Agaricales* species. The result showed that few Pcls were widely distributed indicating that these introns may be inherited from the common ancestors, while the same Pcls were detected in distant species from different lineages providing the evidence of intron loss/gain in the evolution of *Agaricales* species by intron transfer (Huang et al., 2021). This study revealed that Pcl of Ancylistanceae introns varied greatly. Some introns (e.g., P19 and P30) were widely distributed in the *cox1* gene and P6 in the *cob* gene. These introns may be inherited from the ancestors of Ancylistanceae. This phenomenon indicated the potential transfer of introns in the evolution of Ancylistanceae. However, the intronic comparative analysis was conducted within only 10 Ancylistanceae mitogenomes, so the intron loss/gain events were absent of strong evidences.

Repetitive elements in six Conidiobolus s.s. mitogenomes were obtained by BLAST analysis against themselves, resulting in a length variation of 0.2–5.8%. Strangely, *C. chlamydosporus* mitogenome was detected in most tandem repeats (50), while it possessed the smallest mitogenome size among six *Conidiobolus* s.s. mitogenomes. With BLAST to their nuclear genomes, 62–150 aligned fragments were examined between nuclear and mitochondrial genomes of the six *Conidiobolus* s.s. species. This result indicated that the natural gene fragment transfer events may have occurred between mitogenomes and nuclear genomes during the evolution of six *Conidiobolus* s.s. species.

Traditionally, the ITS, LSU rDNA sequences, and morphological characteristics were widely used in species identification and evolution (Zheng et al., 2007; Zhao et al., 2021; Zong et al., 2021). In addition, single- or multi-gene loci, SSRs, and single-nucleotide polymorphisms (SNPs) have been widely used to study the evolution and polymorphism of fungi during the last two decades (Tsykun et al., 2017). Phylogenetic analyses based on multi-gene loci and even whole genomic data developed rapidly as the cost of sequencing decreased (Spatafora et al., 2016; Nie et al., 2019). Especially, SNPs called from whole genome were extensively used to study polymorphisms in fungi (Duan et al., 2018; Ju et al., 2020), and SNP-based genome-wide association studies (GWAS) were widely used to explore the control mechanism for wheat leaf rust (Liu F. et al., 2020).

The genus *Conidiobolus* s.l. was divided into four clades representing four genera: *Capillidium*, *Microconidiobolus*, *Neoconidiobolus*, and *Conidiobolus* s.s. based on the multiple genetic locus phylogeny (Nie et al., 2020b). Phylogenomic analysis based on the 14 PCGs of mitogenomes revealed that six *Conidiobolus* s.s. species were grouped together with three related species: *C. heterosporum*, *M. nodosus*, and *N. thromboides* in the *Entomophthoromycotina* lineage. This phylogram was consistent with our previous study and the phylogenomic analysis based on the 192 orthologous proteins of nuclear genomes (Spatafora et al., 2016; Nie et al., 2019). Also, this phylogram revealed that four lineages among 10 *Conidiobolus* s.l. species demonstrated that 14 mitochondrial PCGs are suitable to clarify phylogenetic relationships among this fungal groups.

In our previous study, the complete mitogenome of *C. heterosporum* (synonym with *Conidiobolus heterosporus*) has been reported, and the comparative analysis with other 22 mitogenomes of basal fungi revealed a high variation in mitogenomes sizes, gene order, GC content, genetic code, etc. (Nie et al., 2019). It was worth to note that the horizontal transfer event of *C. heterosporum* introns has failed to detect strong evidence due to rarely available mitogenomes of basal fungi. In this study, we have sequenced, assembled, and annotated six additional mitogenomes of basal fungi in the genus *Conidiobolus* s.s., and the comparative analysis has also been provided to give a comprehensive evolution of mitogenomes in six *Conidiobolus* s.s. species. The result confirmed that the number and length of intron were the main factors contributing to size variations of *Conidiobolus* s.s. mitogenomes and confirmed our previous taxonomical treatment of *Conidiobolus* s.l. Meanwhile, the comparison within six *Conidiobolus* s.s. mitogenomes showed high intronic variations and gene rearrangement. However, the evidence of intron loss/gain events needs further comparative analysis by using more mitogenomes in the family Ancylistanceae, even in the order Entomophthorales. Fortunately, more and more reported mitogenomes of basal fungi will help us to promote the understanding of its evolution and phylogeny in the future. In addition, mitogenomic sequences from a sufficient number of individuals within a certain species can play essential potential in population heredity, pan-genome, and species definition (Duan et al., 2018; McCarthy and Fitzpatrick, 2019; Nie et al., 2019).

## Conclusion

We have sequenced, assembled, and annotated six *Conidiobolus* s.s. mitogenomes. The result confirmed that the number and length of introns were the main factors contributing to size variations of *Conidiobolus* s.s. mitogenomes. Phylomitogenomics backed up our previous taxonomical treatment of *Conidiobolus* s.l., i.e., dividing into four genera. Comparative analysis within *Conidiobolus* s.s. mitogenomes showed a high gene synteny along with deep intronic variations and gene rearrangement. Potential intron loss/gain and transfer events occurred during the evolution of the family Ancylistanceae.

## Data Availability Statement

The datasets presented in this study can be found in online repositories. The names of the repository/repositories and accession number(s) can be found in the article/Supplementary Material.

## Author Contributions

BH and XL conceived and designed the experiments. YN, HZ, ZW, and ZZ analyzed the data. YN, XL, and BH wrote the manuscript. All authors read and approved the final manuscript.

## Conflict of Interest

The authors declare that the research was conducted in the absence of any commercial or financial relationships that could be construed as a potential conflict of interest.

## Publisher’s Note

All claims expressed in this article are solely those of the authors and do not necessarily represent those of their affiliated organizations, or those of the publisher, the editors and the reviewers. Any product that may be evaluated in this article, or claim that may be made by its manufacturer, is not guaranteed or endorsed by the publisher.

## Footnotes

1 http://www.indexfungorum.org/, accessed on September 1, 2021.

2
https://www.ncbi.nlm.nih.gov/genome/

3
https://genome.jgi.doe.gov

4 https://www.ncbi.nlm.nih.gov, accessed on May 1, 2021.

5
http://megasun.bch.umontreal.ca/cgi-bin/mfannot/mfannotInterface.pl

6
http://www.ncbi.nlm.nih.gov

7
http://www.dnastar.com/

8
http://archive.gramene.org/db/markers/ssrtool

## Supplementary Material

Supplementary Figure 1 Position class (Pcl) analysis of nine conserved protein-coding genes (PCGs) within 10 *Conidiobolus* s.l. species. The phylogenetic tree is constructed by maximum-likelihood (ML) method based on 14 concatenated mitochondrial core proteins.

Supplementary Figure 2 Codon usage analyses of *Conidiobolus* s.l. mitogenomes. **(A)** *C. chlamydosporus*, **(B)** *C. lichenicolus*, **(C)** *C. mycophagus*, **(D)** *C. polyspermus*, **(E)** *C. polytocus*, **(F)** *C. taihushanensis*, **(G)** *Capillidium heterosporum*, **(H)** *Conidiobolus* sp., **(I)** *Microconidiobolus nodosus*, and **(J)** *Neoconidiobolus thromboides*.
