# Supplementary figures and images for: The Gene Rearrangement, Loss, Transfer, and Deep Intronic Variation in Mitochondrial Genomes of Conidiobolus
Source: Front Microbiol. 2021 Nov 11;12:765733. doi: 10.3389/fmicb.2021.765733 (PMC8632527; doi:10.3389/fmicb.2021.765733)

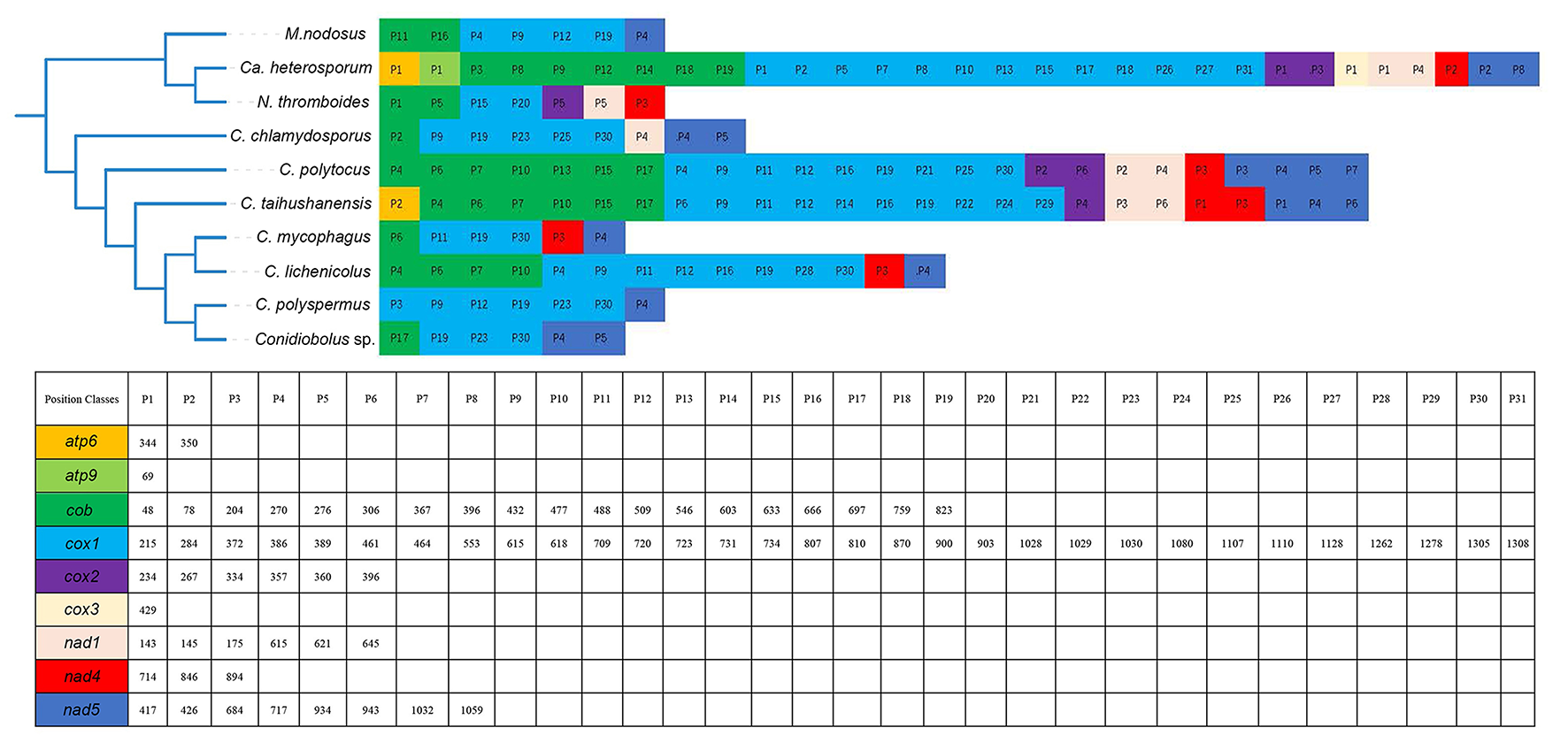

Supplement: Supplementary Figure 1 — Position class (Pcl) analysis of nine conserved protein-coding genes (PCGs) within 10 Conidiobolus s.l. species. The phylogenetic tree is constructed by maximum-likelihood (ML) method based on 14 concatenated mitochondrial core proteins. [file Image_1.TIF]

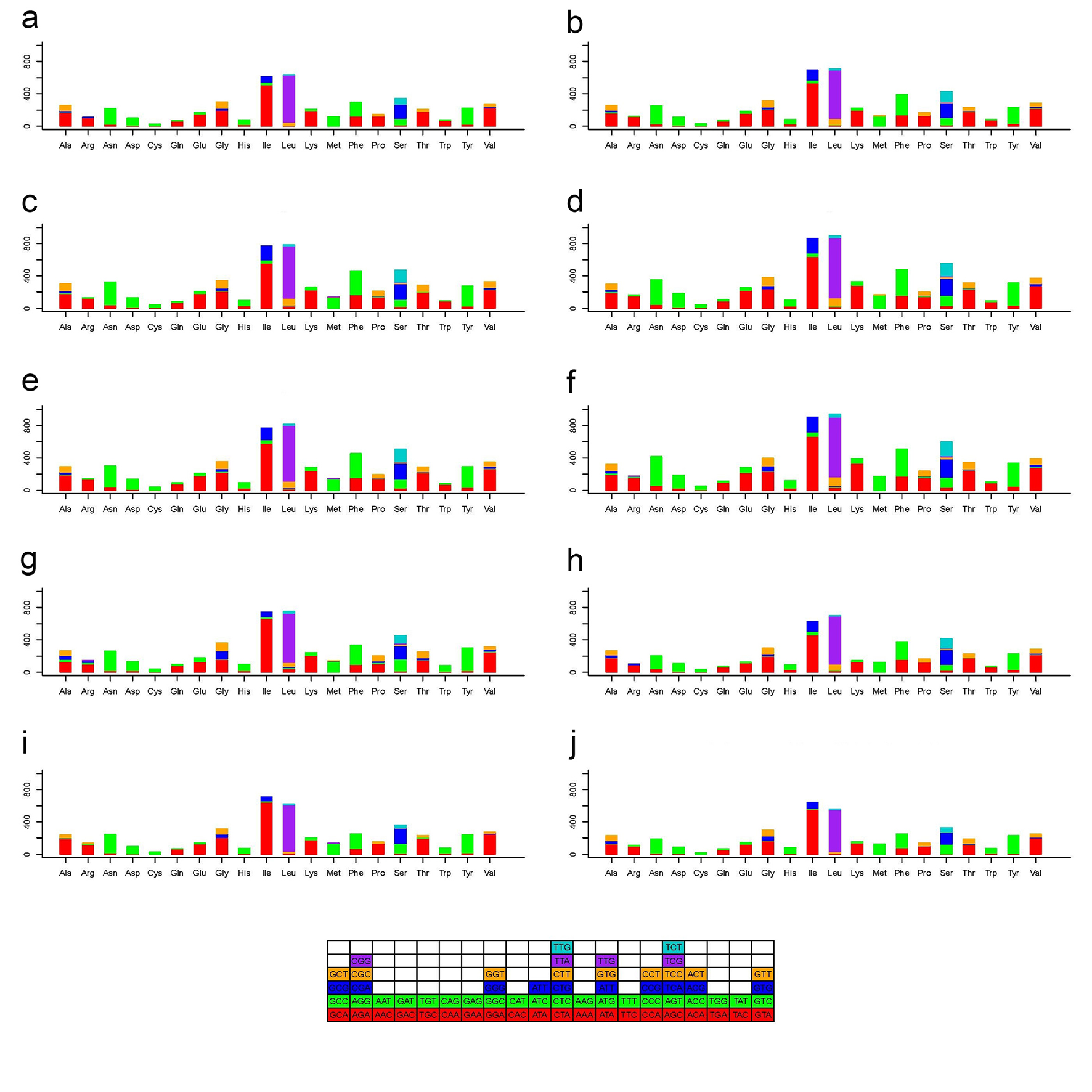

Supplement: Supplementary Figure 2 — Codon usage analyses of Conidiobolus s.l. mitogenomes. (A) C. chlamydosporus, (B) C. lichenicolus, (C) C. mycophagus, (D) C. polyspermus, (E) C. polytocus, (F) C. taihushanensis, (G) Capillidium heterosporum, (H) Conidiobolus sp., (I) Microconidiobolus nodosus, and (J) Neoconidiobolus thromboides. [file Image_2.TIF]
